# Genetic similarity of circulating and small intestinal virus at the end stage of acute pathogenic simian-human immunodeficiency virus infection

**DOI:** 10.3389/fmicb.2013.00204

**Published:** 2013-07-22

**Authors:** Megumi Matsuyama-Murata, Katsuhisa Inaba, Reii Horiuchi, Yoshinori Fukazawa, Kentaro Ibuki, Masanori Hayami, Tomoyuki Miura

**Affiliations:** Laboratory of Primate Model, Experimental Research Center for Infectious Diseases, Institute for Virus Research, Kyoto UniversityKyoto, Japan

**Keywords:** AIDS, HIV, SIV, SHIV, rhesus, phylogenesis, evolution, immunohistochemistry

## Abstract

To understand the pathogenicity of acquired immune deficiency syndrome (AIDS), it is important to clarify where, when and how the virus replicates in the body of infected individuals. To identify the major virus replication site at the end stage of SHIV infection, we investigated the systemic tissues of SHIV-infected monkeys that developed AIDS-like disease. We quantified proviral DNA, and compared the mutation patterns of the viruses in various systemic tissues and in peripheral blood through phylogenetic analysis of the full genome sequence. We found that the amounts of proviral DNA detected in internal tissues were higher than those in peripheral blood mononuclear cells. In the sequence and phylogenetic tree analyses, the mutation patterns of the viruses in each tissue were generally different. However, the mutation pattern of the viruses in the jejunum and mesenteric lymph node were most similar to that of plasma viral RNA among the tissues examined in all three monkeys. In two of the three monkeys, which were euthanized earlier, viruses in the jejunum and mesenteric lymph node occupied the root position of the phylogenetic tree. Furthermore, in these tissues, more than 50% of SHIV-expressing cells were identified as macrophages based on co-expression of CD68. These results suggest that macrophages of the small intestine and/or mesenteric lymph node are the major virus production site at the end stage of SHIV infection of macaques.

## Introduction

To understand the pathogenicity of AIDS, it is important to clarify where, when and how HIV replicates in the body of infected individuals. However, there are limitations to analysis of systemic human tissues. Animal models can substitute for humans; i.e., the simian immunodeficiency virus (SIV)-macaque model. SIV-infected macaques develop AIDS-like disease at an interval from several months to years (Desrosiers, 1990). However, SIV is closely related to HIV-2, but not HIV-1; in particular, the immune response of SIV *env* is thought to be different from the immune response of HIV-1 (Overbaugh et al., 1991).

Another animal model is that comprising simian-human immunodeficiency virus (SHIV) and the macaque. SHIVs are chimeric viruses that contain HIV-1 *env*, *rev*, *tat*, and *vpu* genes on a background of SIVmac, and some strains of SHIV cause AIDS-like disease (Reimann et al., 1996, 1999; Joag et al., 1997; Harouse et al., 1998). One such virus is SHIV-KS661, which is characterized by the profound depletion of CD4+ T cells and maintenance of high viral loads (Fukazawa et al., 2008). The SHIV-macaque model has two main merits. First, SHIVs contain HIV-1 *env, rev, tat*, and *vpu* genes (Kuwata et al., 1995), and it is especially important that the *env* gene is from HIV-1. SHIVs are reported to include both CXCR4-tropic and CCR5-tropic strains; CXCR4-SHIV targets the peripheral blood and thymus, whereas CCR5-SHIV targets gut-associated lymphoid tissues (Harouse et al., 1999; Ho et al., 2005), although most known SIV strains are CCR5-tropic. Therefore, this would be a useful approach to understanding the cell tropism and pathogenesis of the *env* gene of HIV-1. The second advantage of using the SHIV-macaque model is that it is easy to evaluate pathogenesis because the disease progresses more quickly in CXCR4-SHIV-infected macaques compared to SIV-infected macaques. SHIV-infected macaques develop CD4+ T cell depletion within several weeks, and acute AIDS-like disease from several weeks to months (Joag et al., 1996; Reimann et al., 1996).

Previous studies using the SIV-macaque model of AIDS have demonstrated that SIV replicates rapidly around the intestine during primary infection, within a couple of weeks post-infection (Veazey et al., 1998; Li et al., 2005; Mattapallil et al., 2005). Moreover, CD4+ T cell depletion upon primary infection with HIV-1 has been reported to occur in the gastrointestinal tract (Meng et al., 2000; Brenchley et al., 2004; Mehandru et al., 2004). However, the dynamic states of the virus during other phases, especially the AIDS stage, have not been defined.

During primary infection of SIV or SHIV, the virus-producing site has been defined by quantification of proviral DNA and viral RNA using quantitative PCR and plaque assay in systemic tissues (Couedel-Courteille et al., 1999, 2003; Miyake et al., 2004). However, because the viruses were detected in systemic tissues at high levels during the AIDS stage, it was difficult to identify the site of virus production by only quantification of proviral DNA and viral RNA. Therefore, we focused on the nucleotide mutations of virus in systemic tissues and in peripheral blood, and examined the viral dynamics in SHIV-infected macaques at the end stage. Previous studies have analyzed the viral sequences in several tissues of infected individuals and suggested that HIV and SIV evolve differentially in those tissues (Kodama et al., 1993; Wong et al., 1997; Oue et al., 2013). However, they did not define the major site of virus production because analyzed tissues were limited. Based on previous reports, we hypothesized that the site of virus replication might be determined by phylogenetic analyses. In this study, direct sequencing of the full SHIV genome was performed to detect mutations, and a consensus sequence was determined for each tissue and for peripheral blood. Then, phylogenetic analyses were performed to compare the virus sequence patterns among tissues. Furthermore, we conducted an immunohistochemical analysis of virus-producing cells. Based on these analyses, we identified the major virus replication site at the end stage of SHIV infection.

## Materials and methods

### Viruses

We used the molecular clone viruses, SHIV-KS661 (GenBank accession no. AF217181) and SHIV-KS705. SHIV-KS661 was constructed from the consensus sequence of SHIV-C2/1. SHIV-C2/1 was generated by *in vivo* passage in cynomolgus monkey (*Macaca fascicularis*) of SHIV-89.6 (containing *env*, *tat*, *rev* and *vpu* gene of HIV-1) (Shinohara et al., 1999).

SHIV-KS661 causes rapid and profound depletion of CD4+ T cells. In this study, the SHIV-KS661 virus stock was prepared by two methods. In method 1, SHIV-KS661 was prepared from culture supernatants of COS-1 cells by direct transfection with a molecular clone. In method 2, it was prepared from the supernatants of a human cell line, CEMX174 by infection with the virus of method 1. SHIV-KS705 is a chimeric virus of SHIV-KS661 and SHIV-#64, and contains the *env* gene of SHIV-KS661 (SphI ~ XhoI fragment) in a SHIV-#64 background. SHIV-#64 was a molecular clone derived from SHIV-89.6P, which was generated by *in vivo* passage of SHIV-89.6 in rhesus monkeys (*Macaca mulatta*) (Kozyrev et al., 2001). The SHIV-KS705 stock was prepared from the supernatant of culture of a human cell line, M8166 by infection with the virus of method 1. There was no serious influence of the virus preparation on the results.

### Monkeys and inoculation

Three Indian rhesus macaques were used. Throughout the experimental period, the monkeys were treated in accordance with the institutional regulations approved by the Committee for Experimental Use of Non-human Primates at the Institute for Virus Research, Kyoto University. Monkey MM273 was inoculated intravenously with 1.2 × 10^4^ TCID_50_ SHIV-KS661; MM376 was inoculated intrarectally with 2000 TCID_50_ SHIV-KS661, and MM340 was inoculated intravenously with 10^4^ TCID_50_ SHIV-KS705. All three monkeys (MM340, MM376 and MM273) developed AIDS-like disease and were euthanized at 22, 33, and 90 weeks post-inoculation (wpi), respectively.

### Sample collection

Blood was collected under ketamine anesthesia. After the separation of plasma, peripheral blood mononuclear cells (PBMC) were separated from anticoagulated blood with Lymphocyte Separation Solution (Nacalai Tesque, Kyoto, Japan) by density gradient centrifugation. Plasma and PBMC were frozen at −80°C until analysis. Intravenous pentobarbital (Nembutal; Abbott Laboratories, Abbott Park, IL) (40 mg/kg) was administered for deeper anesthesia. Following thoracotomy, the right atrium was incised, and 1000 ml of sterile heparinized saline (5 U/ml) was infused into the left ventricle using an 18-gauge needle attached to infusion tubing. Following perfusion, systemic organs were obtained. Organ samples were frozen at −80°C until used for quantification of proviral DNA.

### Quantification of plasma viral RNA

Virion-associated SHIV RNA loads in plasma were measured by real-time reverse transcription (RT)-PCR assay (Suryanarayana et al., 1998; Motohara et al., 2006). Briefly, total RNA was prepared from plasma (140 μl) of each monkey using a QIAamp Viral RNA Kit (Qiagen, Hilden, Germany). RT reactions and PCR were performed using the Platinum® qRT-PCR ThermoScript™ One-Step System (Invitrogen, Carlsbad, CA) for the SIV *gag* region using the following primers: SIV2-696F (5′-GGA AAT TAC CCA GTA CAA CAA ATA GG-3′) and SIV2-784R (5′-TCT ATC AAT TTT ACC CAG GCA TTT A-3′). A labeled probe, SIV2-731T (5′-Fam-TGT CCA CCT GCC ATT AAG CCC G-Tamra-3′), was used for detection of the PCR products. These reactions were performed using a Prism 7700 Sequence Detector (Applied Biosystems, Foster City, CA) and analysed using the manufacturer's software. A standard curve was generated from dilutions whose copy numbers were known, and the RNA in the plasma samples was quantified based on this standard curve.

### Quantification of proviral DNA

The proviral DNA loads in tissues were determined by quantitative PCR, as described previously (Motohara et al., 2006). DNA samples were extracted directly from frozen tissues using a DNeasy® Tissue Kit (Qiagen). PCR was performed with Platinum® Quantitative PCR SuperMix-UDG (Invitrogen) using the same primer set and probes that were used for RT-PCR. A standard curve was generated from a plasmid DNA sample containing the full genome of SHIV-KS661, which was quantified using a UV-spectrophometer. Tissue DNA samples were also quantified using a UV-spectrophometer to use 1 μg for each reaction and detection limit of this assay was 10 copies/μg.

### Flow cytometry

The frequency of CD4-positive T cells in peripheral blood was examined by flow cytometry. Lymphocytes were treated with anti-CD3 (FN-8-FITC; Biosource, Camarillo, CA) and anti-CD4 (Nu-TH/I-PE; Nichirei, Tokyo, Japan) monoclonal antibodies and examined using a FACScan analyser (Becton Dickinson Biosciences). The absolute number of lymphocytes in the blood was determined using an automated blood cell counter (F-820; Sysmex, Kobe, Japan).

### Sequence analysis

Total DNA was extracted from the cell pellet with a DNeasy® Tissue Kit. Total RNA was extracted from the cell pellet with a RNeasy Protect Kit (Qiagen). RNA extraction was performed in the condition adding DNase I to exclude the possibility of contamination of proviral DNA into RNA. Virion RNA was prepared from plasma using a QIAamp Viral RNA Kit (Qiagen). Seventy-four consensus primers were synthesized for every 250–300 bp throughout the genome of SHIV for full genome sequencing of both the forward and reverse strands. Using these primers with RNA samples, RT-PCR was performed using the One-step RT-PCR Kit (Qiagen). For DNA samples, PCR was performed using Platinum® PCR SuperMix (Invitrogen). The PCR products were purified using QIAquick Spin (Qiagen) and direct sequencing was carried out using the ABI PRISM® Big Dye® Terminator v1.1 Cycle Sequencing Kit (Applied Biosystems) with an automated sequencer (ABI PRISM 310 genetic analyser; PerkinElmer, Emeryville, CA).

### Phylogenetic analysis

Sequence alignments were performed using the ClustalW program (Thompson et al., 1997). Final tree topologies were visualized with Tree View.

### Immunohistochemical analysis

The tissue samples obtained from sacrificed monkeys were fixed in 4% paraformaldehyde (PFA)-PBS (1× phosphate-buffered saline) at 4°C overnight, and embedded in paraffin wax. For immunohistochemistry, 4-μm sections were rehydrated and processed for 10 min in an autoclave in 10 mM citrate buffer (pH 6.0) to unmask the antigens. The samples were treated sequentially with TBS (Tris-buffered saline)-0.05% Tween 20 and 3% aqueous hydrogen peroxide. Anti-SIV Nef mouse monoclonal antibody (FIT Biotech, Tampere, Finland) reactions were conducted at 4°C overnight. After washing with TBS-0.05% Tween 20, sections were incubated at room temperature for 1 h with Envision+ Kit, a horseradish peroxidase-labeled anti-mouse immunoglobulin polymer (DAKO Corp., Carpinteria, CA). Subsequently, the reaction was visualized using diaminobenzidine substrate (DAKO) as a chromogen at room temperature for 5 min. Samples were then rinsed in distilled water and counterstained with hematoxylin.

To detach the primary antibody from enzymatic-immunostained sections, those sections were washed three times in 0.2 M glycine (pH 2.2) buffer for 2 h each at room temperature. The sections were processed for 10 min in an autoclave in 10 mM citrate buffer (pH 6.0), and were then incubated with anti-human CD3 rabbit polyclonal antibody (DAKO) and anti-human CD68 mouse monoclonal antibody (DAKO) at room temperature. Anti-rabbit IgG antibody-labeled Alexa 488 and anti-mouse IgG antibody-labeled Alexa 594 were used as secondary antibodies. Multiple staining samples were observed with a Leica confocal microscope.

## Results

### Infection of macaque monkeys with SHIV-KS661 or SHIV-KS705

**Figure 1:**
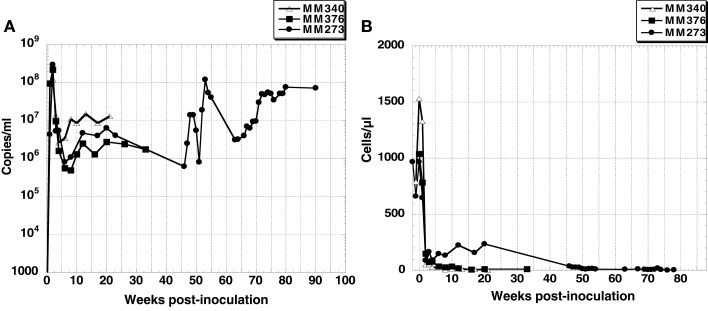
**(A)** Plasma viral RNA load and **(B)** CD4+ T cell count in peripheral blood of three SHIV-inoculated monkeys. Monkey MM340 inoculated with SHIV-KS705 was euthanized at 22 wpi; MM376 and MM273 inoculated with SHIV-KS661 were euthanized at 33 wpi and 90 wpi, respectively.

Two of three macaque monkeys, MM273 and MM376, were inoculated intravenously and intrarectally with SHIV-KS661, respectively. The other macaque monkey, MM340, was inoculated intravenously with SHIV-KS705. In all monkeys, plasma viral RNA increased rapidly after inoculation, and reached peak levels of ~10^8^ copies/ml at 2 weeks post-inoculation (wpi). After that, plasma viral RNA decreased to ~10^6^ to 10^7^ copies/ml and was maintained at >10^6^ copies/ml (Figure 1A). The number of CD4+ T cells declined to ~200 cells/μl at 3 weeks after inoculation, and remained at zero level throughout the infection in MM376 and MM340, whereas in MM273, the number of CD4+ T cells recovered to ~300 cells/μl from 12 to 20 wpi, but after 46 wpi, it maintained a zero level (Figure 1B). All monkeys showed acute diarrhea and had lost weight at the time of euthanasia. MM340, MM376, and MM273 were euthanized at 22, 33, and 90 wpi, respectively.

### Quantification of proviral DNA in systemic tissues

**Figure 2:**
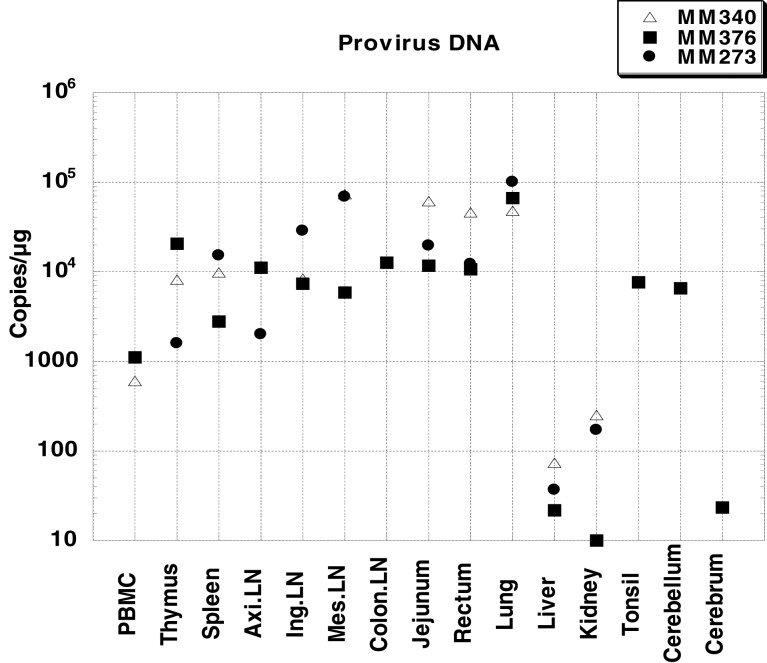
**Proviral DNA loads in systemic tissues of three SHIV-inoculated monkeys.** Viral loads were determined by quantitative PCR and are expressed as viral DNA copy numbers per microgram of total DNA extracted from the tissue homogenates. PBMC was not collected from MM273. Axillary LN was not collected from MM340. Colon LN, cerebellum, cerebrum, and tonsil were collected only from MM376.

Proviral DNA in systemic tissues was quantified by quantitative PCR (Figure 2). In all three monkeys, the proviral DNA was detected at high levels in the intestinal tract (jejunum and rectum), as well as lymph nodes (axillary LN, inguinal LN, mesenteric LN and colon LN); the proviral DNA level in lung was especially high (10^5^ copies/μg). The level of proviral DNA in PBMC was 10^3^ copies/μg, which was lower than that in other tissues. In monkey MM376, high titers of proviral DNA were detected in the cerebellum. These results showed that the virus infected various systemic tissues at higher levels than that in PBMC during the AIDS stage.

### Sequence analysis

To compare the genotypes of viruses in systemic tissues and in peripheral blood, direct sequencing was performed, covering the whole genome of the viral RNA extracted from plasma and proviral DNA extracted from various tissue samples. In systemic tissue samples from MM340, 16 mutations and one nucleotide deletion were detected in the whole genome of SHIV-KS705 (Table 1). Except for one mutation in the *env* gene (8816th nucleotide), all of the mutations were associated with amino acid substitutions. Most mutations were detected in the spleen. For MM376, 30 mutations were detected in the whole genome of SHIV-KS661 (Table 2). Most mutations were accumulated in the rectum. MM340 and MM376, which were euthanized earlier, showed more mutations in systemic tissues than in peripheral blood such as plasma and PBMC, and these occurred in the region of the envelope gene. For MM273, 90 mutations were observed in the whole genome of SHIV-KS661. Almost all mutations were associated with amino acid substitutions. In axillary LN, few mutations were observed in the region of *env*-gp120. Also in MM273, the number of mutations observed in peripheral blood was the same as that in the various tissues. To compare the mutation patterns of produced viruses with those of proviruses, viral RNA in tissues of MM273 was sequenced. In the jejunum and mesenteric LN, the regions of mutations in virus RNA and provirus DNA were almost identical (Table 3). This suggested that the viruses produced in tissues are the same as the provirus DNA in the infected cells. In all monkeys, the mutation patterns of viruses differed among tissues; in the monkey that was euthanized earlier, the mutations were limited, whereas more mutations had accumulated in the monkey euthanized later.

**Table 1:** **Consensus nucleotide mutations in MM340**.

**Nucleotide position**	**KS705**	**Plasma**	**PBMC**	**Thymus**	**Spleen**	**Ing.LN**	**Mes.LN**	**Rectum**	**Jejunum**	**Lung**	**Amino acid change**	**Genomic region**
312	T	C	C	C	C	C	C	C	C	C		PBS
323	T				A	A						
543	T			C							V/A	gag-MA
569	A	G	G	G	G	G	G	G	G	G	K/E	
638	G	A	A	A	A	A	A	A	A	A	A/V	
2948	C	T	T		T	T	T		T		L/S	RT
5165	C	T			T		T		T		P/S	vif
5190	G										R/K	
6838	T				G						I/R	env gp120
6859	A							G			E/G	
6906	A			G							S/G	
7272	A			G	G	G					I/V	
8624	C				A	A					C/stop	env gp41
											P/T	rev
8803	A			G							E/G	env gp41
											R/G	nef
8816	C	T	T		T	T	T	T	T		A/A	env
											P/L	nef
9359	A	G		G			G	G	G		Y/C	U3
9644 ~ 9		1bp-del		1bp-del					1bp-del			

**Table 2:** **Consensus nucleotide mutations in MM376**.

**Nucleotide position**	**KS661 original**	**Plasma**	**PBMC**	**Thymus**	**Spleen**	**Axi.LN**	**Ing.LN**	**Mes.LN**	**Colon. LN**	**Jejunum**	**Rectum**	**Lung**	**Cerebellum**	**Tonsil**	**Amino acid change**	**Genomic region**
135	T											C				LTR
543	T											C			V/A	
569	A	G	G	G	G	G		G	G	G	G		G	G	K/E	
573	C										T				A/V	
638	G	A	A	A	A	A	A	A	A	A	A	A	A	A	V/I	gag-MA
737	G														V/M		
752	G				A										E/K	
1048	A														V/V	
4644	A												G		Q/Q	pol
5129	A	G			G	G		G	G	G	G	G	G		N/D	vif
5249	T				C					C	C	C	C		Y/H	
5250	A						G		G					G	Y/C	
6186	C				T	T	T	T	T		T	T		T	P/L	vpu
6804	A				G	G			G		G		G		I/V	env gp120
6841	G													A	R/K	
6859	A			G											E/G	
6922	G										A				R/K	
7648	C				A		A	A			A		A	A	T/K	
8095	G										C	C			R/T	env gp41
8109	A				C									C	M/L	
8238	G	A	A		A	A	A	A	A	A	A	A		A	D/N	
8326	A										C	C			K/T	
8576	A										G			G	Q/R	tat
															S/G	rev
															S/S	env gp41
8598	A	G			G	G	G	G		G		G			Y/C	rev
															I/V	env gp41
8624	C				A							A			P/T	rev
															C/stop	env gp41
9011	G											A			R/K	nef
9263	C			T							T				S/L	nef/U3
9275	T										C				I/T	
9281	A	T	T		T	T	T	T	T	T		T	T	T	T/F	
9359	A	G	G	G		G	G	G	G	G	G	G	G		Y/C	

**Table 3:** **Consensus nucleotide mutations in MM273**.

**Nucleotide position**	**KS661 original**	**Plasma**	**Thymus**	**Spleen**	**Axi.LN**	**Ing.LN**	**Mes.LN**	**Mes.LN RNA**	**Jejunum**	**Jejunum RNA**	**Rectum**	**Lung**	**Amino acid change**	**Genomic region**
107	G										A	A		LTR
135	T	C		C			C	C	C	C	C	C		
543	T	C	C	C	C	C	C	C	C	C	C	C	V/A	gag-MA
569	A	G	G	G	G	G	G	G	G	G	G	G	K/E	
572	G	A	A	A	A	A	A	A	A	A	A	A	A/I	
638	G		A	A	A	A	A	A	A	A	A	A	V/I	
969	T	C	G	G		C	C	C	C	C		C	V/A	gag-CA
1016	A	G					G	G	G	G			I/V	
1532	C	A	A	A	A	A	A	A	A	A	A	A	L/I	
2453	A	G	G	G	G	G	G	G	G	G	G	G	K/R	pol-RT
4172	C	T	T	T	T	T	T	T	T	T	T		A/V	pol-INT
4300	C		G	G		G	G						T/A	
4478	A		T			T							H/L	
4664	A					T							S/L	
5057	A	G	G	G	G	G	G	G	G	G	G	G	K/E	vif
5072	A	G	G	G	G	G	G	G	G	G		G	T/A	
5238	G		A	A				A				A	R/K	
5249	T	C		C			C	C	C	C	C	C	Y/H	
5250	A		G	G	G	G							Y/C	
5918	G	A	A	A	A	A	A	A	A	A	A	A		vpr
6040	C											T	Q/P	tat
6126	A			C	C			C				C		
6146	G	A	A	A	A	A	A	A	A	A	A	A		
6328	A								G				A/E	vpu
6363	C	A				A	A	A	A	A	A			
6394	C				T									
6553	A		G	G	G							G	H/R	
6766	T	C				C	C	C					L/P	
6772	G	A	A	A							A		S/N	
6788	G					A							M/I	
6843	A	G	G	G		G	G	G	G	G	G	G	N/D	
6846	A		G	G		G						G	K/E	
6850	T	C					C	C	C	C	C		V/A	
6877	G					T							R/I	
6880	T		A										L/Q	
6890	A					G								
6893	A	G	G				G		G	G	G			
6897	A	G				G	G	G	G		G		K/E	
6907	G	A				A	A	A	A	A	A		S/N	
6936	A			G		G	G					G	N/D	
7003	T	C	C	C		C	C	C	C	C	C	C	V/A	
7182	G	A	A	A		A	A	A	A	A	A	A	D/N	
7251	A		T	T								T	N/Y	
7269	T					C							S/P	env-gp120
7284	A					G							R/G	
7306	A	G	G	G		G	G	G	G	G			N/S	
7385	A											T		
7402	T		A	A		A							F/Y	
7464	A	C	C	C			C	C	C	C	C	C	S/R	
7465	G	A	A	A		A	A	A	A	A	A	A	S/Q	
7533	G	A	A	A		A	A	A	A	A	A	A	A/T	
7554	G	A	A	A			A	A	A	A	A		E/K	
7647	A	G	G	G		G	G	G	G	G	G	G	T/A	
7707	A			G					G		G		N/R	
7711	G	T		A		T	A	T			A		S/IorN	
7725	A	C	C	C	C	C	C	C	C	C	C	C	T/P	
7831	G								A	A			R/K	
7847	A	G	G	G	G	G	G	G	G	G	G	G		
8109	A					T							M/V	
8134	A	G	G	G	G	G	G	G	G	G	G	G	K/R	
8238	G	A	A	A	A	T	A	A		A		A	D/N	
8352	G											A	D/N	
8431	C					T							A/V	
8453	A	G	G	G	G	G	G	G	G	G	G	G		
8492	C	T			T		T	T	T	T	T			
8493	C	T	T	T	T	T	T	T	T	T	T	T	L/F	
8587	C	T	T	T	T	T	T	T	T	T	T	T	S/F	env-gp41
8605	C	T	T		T	T	T	T	T	T	T	T	A/V	
8613	C		T	T		T	T					T	R/W	
8734	A	T	T	T	T	T	T	T	T	T	T	T	Q/L	
8743	G		A										S/N	
8759	T	G	G	G	G	G	G	G	G	G	G	G	N/K	
8780	A	G		G	G	G	G	G	G	G	G	G		
8792	C		T										A/V	nef
8803	A											G	R/G	
9011	G	A	A	A	A	A	A	A	A	A	A	A	R/K	
9080	T	C	C	C	C	C	C	C	C	C	C	C	V/A	
9082	T	C	C	C	C	C	C	C	C	C	C	C	S/P	
9100	C	A	A	A	A	A	A	A	A	A	A	A	P/T	
9116	G		C										S/T	
9226	G	A				A	A	A	A	A	A		E/K	
9229	G	A	A		A	A	A	A	A	A	A	A	E/K	
9322	A	G					G	G	G	G	G		N/D	
9341	A	G	G	G	G	G	G	G	G	G	G	G		
9380	C			G	G	G							T/S	
9430	C	T	T	T			T	T	T	T	T		P/S	
9460	G	A	A	A	A	A	A	A	A	A	A	A	V/I	
9518	G	A	A	A	A	A	A	A	A	A	A	A	R/K	
9558	G				A									
9661	A	G	G	G	G	G	G	G	G	G	G	G	I/V	
6220–6228		AAT-ins	AAT-ins	AAT-ins	AAT-ins	AAT-ins	AAT-ins	AAT-ins	AAT-ins	AAT-ins	AAT-ins	AAT-ins		vpu
6898–6901		AAA-del					AAA-del	AAA-del	AAA-del	AAA-del	AAA-del			env-gp120(V2)
6757–6775				18bp-del										env-gp120(V1)
6757–6778			21bp-del											env-gp120(V1)
6757–6779												23bp-del		env-gp120(V1)
6908–6916			9bp-del									9bp-del		env-gp120(V2)
6911–6920				9bp-del										env-gp120(V2)
8799–8831		33bp-del		33bp-del		33bp-del	33bp-del	33bp-del	33bp-del	33bp-del	33bp-del			nef
8839–8886			48bp-del											nef

Moreover, in this study, two mutations of *gag*-matrix (569th and 638th nucleotide) were detected in all three macaques, while a single mutation of *nef* (9359th nucleotide) was detected in two of three macaques (Tables 1–3).

### Phylogenetic analysis

**Figure 3:**
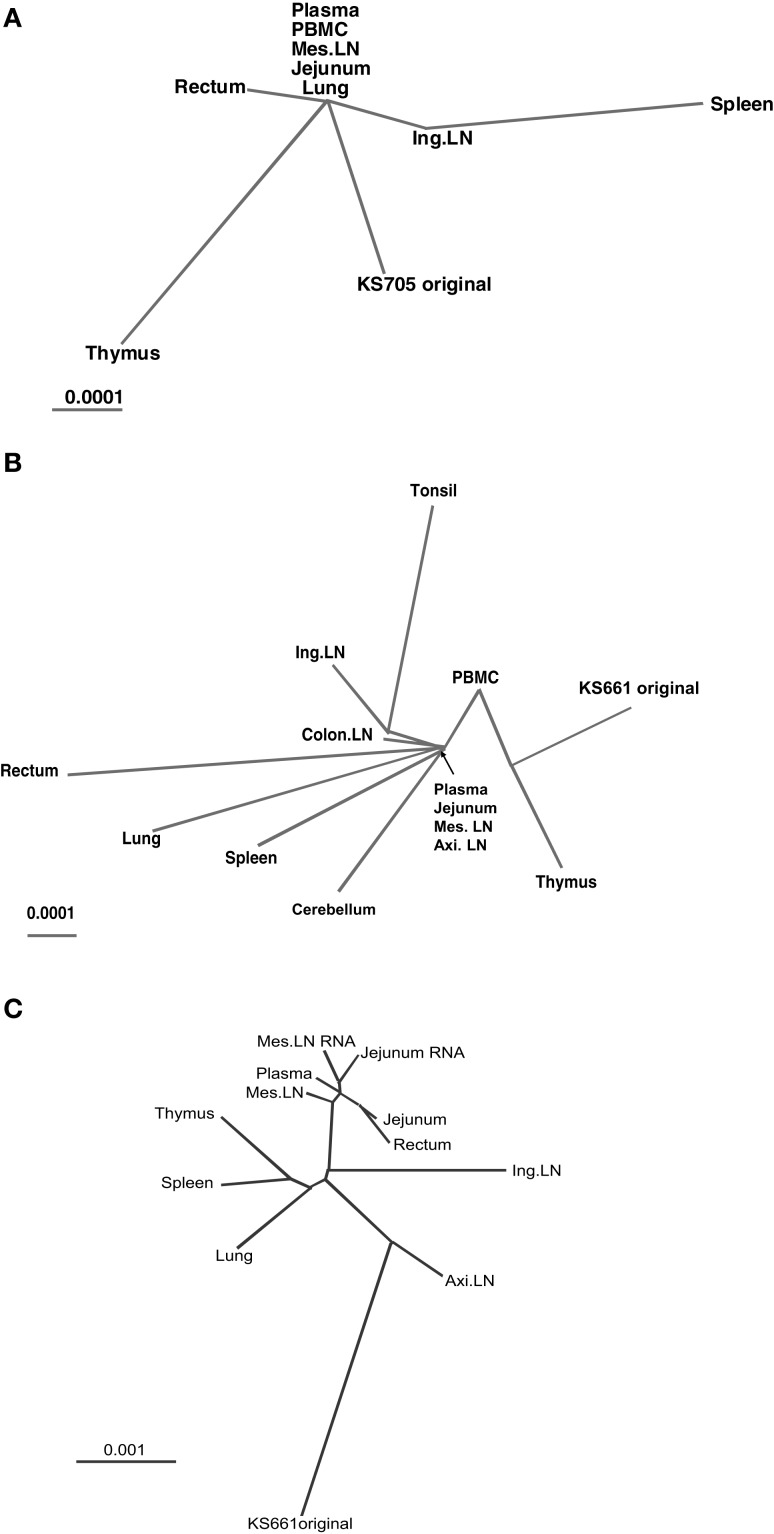
**Unrooted phylogenetic trees of consensus sequences.** Branch length is drawn to scale. **(A)** Phylogenetic tree demonstrating the relationships among consensus sequences from seven tissues and peripheral blood of monkey MM340. **(B)** Phylogenetic tree demonstrating the relationships among consensus sequences from 11 tissues and peripheral blood of MM376. **(C)** Phylogenetic tree demonstrating the relationships among consensus sequences from eight tissues and peripheral blood of MM273.

To clarify the relationships among the genotype of viruses in systemic tissues, phylogenetic analyses were performed. In MM340, the mutations of viruses in systemic tissues and peripheral blood exhibited different patterns, but similarities were noted among viruses in plasma, PBMC, mesenteric LN, jejunum and lung (Figure 3A). In MM376, the mutations of viruses in each tissue and peripheral blood also exhibited different patterns. Similarities were noted among viruses in plasma, jejunum, mesenteric LN, and axillary LN (Figure 3B). In MM273, the viruses accumulated many more mutations in systemic tissues. The mutation patterns among viruses in plasma, rectum, jejunum and mesenteric LN showed greater relatedness than those in other tissues (Figure 3C). In all monkeys, the genotypes of viruses related to the small intestine, such as the jejunum and mesenteric LN, were similar to those of viruses in peripheral blood. In the monkeys that were euthanized earlier (MM340 and MM376), viruses from the jejunum, mesenteric LN and peripheral blood occupied the root position of the phylogenetic tree; whereas in the monkey that maintained a high viral load and was euthanized later (MM273), many mutations accumulated in each tissue independently, but the jejunum, mesenteric LN and peripheral blood formed a cluster. Additionally, virus RNA in the jejunum and mesenteric LN also showed greater similarities with provirus DNA in those tissues (Table 3). Also, in spleen, the mutation pattern of viral RNA was almost identical to that of the provirus DNA (data not shown).

### Immunohistochemical analysis

**Figure 4:**
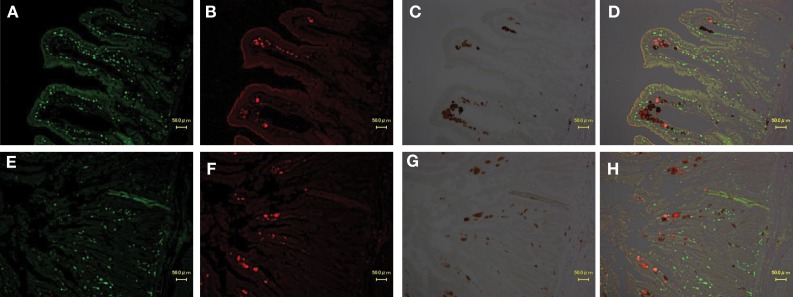
**Identification of virus-producing cells in the jejunum of two macaques (A,B,C,D: MM340, E,F,G,H: MM273).** Immunohistochemical CD3 staining (**A,E**; visualized in green), CD68 (macrophage) staining (**B,F**; visualized in red) and SIV-*nef* (**C,G**; visualized in brown). More than 50% of SIV-*nef*-positive cells were co-localized with CD68+ cells in the merged images (**D,H**).

To identify the type of cells in which viruses replicate in the jejunum and mesenteric LN, an immunohistochemical analysis of CD3+ T cells and CD68+ macrophages in jejunum and mesenteric LN was performed. In all monkeys, >50% of SIV *nef*-expressing cells were judged macrophages according to co-localization of CD68 in jejunum (Figure 4) and mesenteric LN (data not shown). SIV *nef*-expressing CD3+ T cells were rare, and the remaining *nef*-expressing cells were negative for both CD3 and CD68.

## Discussion

In this study, we analysed mutations across the whole SHIV genome in systemic tissues, including the intestine, of SHIV-KS661- or -KS705-infected macaques. Although this study has a limitation as we could analyzed only three macaques, including different viruses and different inoculation route, we think common tendency observed in the three macaques is significant. Previous studies indicated that HIV evolves differentially in various tissues (Delassus et al., 1992; Wong et al., 1997; Buckner et al., 2002; Ritola et al., 2004; Ndolo et al., 2005). Viruses produced from each tissue flow into the blood. Therefore, the majority of the circulating virus population is derived from the major production site. We demonstrated similarities among the mutation patterns of viruses from plasma, jejunum and mesenteric lymph node. This suggests that the major site of virus production at the end stage of SHIV infection of macaques is the small intestine and/or mesenteric LN. Moreover, previous whole-body positron emission tomography (PET) analyses of HIV-1-infected patients and SIV-infected macaques also suggested that the intestine was the target organ at the end stage of HIV-1/SIV infection (Scharko et al., 1996, 2003). Therefore, our finding that the small intestine is the major production site is considered to be a general phenomenon of primate lentivirus infection, and not specific to SHIV.

Concerning primary infection with HIV and SIV, which are transmitted using mainly CCR5 as their co-receptor, some reports suggest affinity for the small intestine. It has been suggested that CD4 lymphocytes of the intestinal tract are depleted selectively during early HIV infection (Clayton et al., 1997). Veazey *et al*. demonstrated that SIV eliminated intestinal CD4+ T cells selectively (Veazey et al., 1998). Other studies demonstrated that SIV replicated exclusively in memory CD4+ T cells and directly destroyed memory CD4+ T cells in the small intestine (Li et al., 2005; Mattapallil et al., 2005). Mesenteric LN harbored viral reservoirs that cause rebound of plasma viremia in SIV-infected macaques upon cessation of combined antiretroviral therapy (Horiike et al., 2012). Another study demonstrated that CXCR4-tropic SHIV decreased peripheral CD4+ T cells, and that CCR5-tropic SHIV decreased intestinal CD4+ T cells (Harouse et al., 1999; Ho et al., 2005). SHIV-KS661 and -KS705 have essentially the same *env* gene and use predominantly CXCR4 as a co-receptor for virus entry (Matsuda et al., 2010; Fujita et al., 2013). However, SHIV-KS661 decreased CD4+ T cells in systemic and mucosal immune tissues during primary infection (Miyake et al., 2006). Macrophage-tropic SHIV use CXCR4, not CCR5, for infections of rhesus macaque peripheral blood mononuclear cells and alveolar macrophages (Igarashi et al., 2003). In this study, we suggested that the small intestine and/or mesenteric LN were the major virus production site at the end stage of SHIV-KS661 and –KS705 infection, regardless of the infection route. These findings suggest that the small intestine is the critical target organ of not only CCR5-tropic HIV/SIV infection, but also CXCR4-tropic SHIV infection.

Phylogenetic analysis suggested that the phylogenetic trees differed among the three macaques. In MM340 and MM376, viruses in the jejunum and mesenteric LN, together with viruses in plasma, occupied the root position of the phylogenetic tree, whereas in MM273, the viral sequences from plasma, jejunum, mesenteric LN and rectum did not occupy the root position, but clustered separately from other tissues. In a phylogenetic tree, viruses in the root position have a consensus mutation pattern. Moreover, viruses in circulating blood tend to have the consensus mutation pattern either because they flow from tissues to the same degree or no particular virus production site is predominant. Therefore, in MM340 and MM376, viruses of the jejunum and mesenteric LN have mutation patterns similar to those in plasma, suggesting that circulating viruses accumulated in and were produced from the small intestine and/or mesenteric LN. On the other hand, in MM273, which retained a high viral load and was euthanized later, the viral sequences from plasma, jejunum, mesenteric LN and rectum did not occupy the root position, but clustered separately from other tissues. This suggests that the viruses in the intestine and/or mesenteric LN were produced mainly in MM273, and that viral production levels from other tissues were relatively low.

Next, we investigated the phenotype of SHIV-producing cells in the small intestine at the end stage of infection. Some studies have suggested that the target cells during the acute phase are CCR5+ memory CD4+ T cells (Li et al., 2005; Mattapallil et al., 2005). However, CD4+ T cells are unlikely to be the target cell since SIV- and SHIV-infected macaques resulted in CD4+ T cell depletion at the end stage of infection (Veazey et al., 2000). Generally, macrophages are regarded as a reservoir because their half-life when infected is substantially longer than that of T cells. As a result, macrophages continue to accumulate HIV-1 and show replication for an extended period, even in patients receiving combined antiretroviral therapy (Sharova et al., 2005). Also, in SHIV-infected macaques, after depletion of CD4+ T cells, macrophages were identified as the virus producing cells (Igarashi et al., 2001). In macaques inoculated with rapid-progressing SIVsmm, the majority of SIV-positive cells in the lymph nodes and gastrointestinal tract were macrophages (Brown et al., 2007). As already reported, macrophages have the potential to produce viruses at the end stage of infection. Our immunohistochemical study also demonstrated that >50% of the SIV *nef*-positive cells were co-localized with CD68-positive cells in the jejunum and mesenteric LN of the three macaques. Because CD68 is expressed in activated cells by phagocytosis and localizes on the membrane of lysosomes, CD68-positive cells can be regarded as macrophages. So, our results are consistent with those findings. Taken together, our data suggest that circulating viruses accumulate and are produced in CD68-positive cells; i.e., macrophages, in the small intestine and/or mesenteric LN. Sequence analyses of virus DNA and RNA extracted from CD68-positive cells isolated from jejunum and mesenteric LN should be investigated to determine which tissue is the real major source of plasma virus in future.

Finally, we detected two mutations of *gag-matrix* (569, 638) in all three macaques, and one mutation of *nef* (9359) in two of three macaques. The fixation of randomly occurring mutants and the emergence of particular variants are caused by selective pressure and the rate of virus turnover (Coffin, 1992). Because common mutations are often detected in SHIV-KS661-infected macaques, these mutations may be important for viral replication *in vivo* at the end stage of SHIV infection. These mutations do not necessarily lead to increased pathogenicity in naïve macaques because these mutants were present in the macaques after the collapse of the immune system (Kuwata et al., 2007). Therefore, we do not think that these mutations may be characterized as association with escape from the immune system. We think they may have a substantial advantage for viral replication in CD68-positive cells; i.e., macrophages. Previous studies about HIV-1 Gag matrix suggested that *gag* targeting and assembly to multivesicular body is important step to produce the virus particles in macrophage (Ono and Freed, 2004). On the other hands, recent study about *nef* mutation demonstrated that some Nef variants were impaired virus replication in monocyte-derived macrophage (Mwimanzi et al., 2011). The significance of these mutations should be investigated further *in vitro* and *in vivo*.

### Conflict of interest statement

The authors declare that the research was conducted in the absence of any commercial or financial relationships that could be construed as a potential conflict of interest.

## References

[B1] Brenchley J. M. Hill B. J. Ambrozak D. R. Price D. A. Guenaga F. J. Casazza J. P. (2004). T-cell subsets that harbor human immunodeficiency virus (HIV) *in vivo*: implications for HIV pathogenesis. J. Virol. 78, 1160–1168 10.1128/JVI.78.3.1160-1168.2004 14722271 PMC321406

[B2] Brown C. R. Czapiga M. Kabat J. Dang Q. Ourmanov I. Nishimura Y. (2007). Unique pathology in simian immunodeficiency virus-infected rapid progressor macaques is consistent with a pathogenesis distinct from that of classical AIDS. J. Virol. 81, 5594–5606 10.1128/JVI.00202-07 17376901 PMC1900277

[B3] Buckner C. M. Gettie A. Tan R. C. Eshetu T. Ratterree M. Blanchard J. (2002). Infection of macaques with a molecular clone, SHIVSF33A2, provides evidence for tissue specific variants. J. Med. Primatol. 31, 164–170 10.1034/j.1600-0684.2002.02002.x 12390538

[B4] Clayton F. Snow G. Reka S. Kotler D. P. (1997). Selective depletion of rectal lamina propria rather than lymphoid aggregate CD4 lymphocytes in HIV infection. Clin. Exp. Immunol. 107, 288–292 10.1111/j.1365-2249.1997.236-ce1111.x 9030865 PMC1904578

[B5] Coffin J. M. (1992). Genetic diversity and evolution of retroviruses. Curr. Top. Microbiol. Immunol. 176, 143–164 10.1007/978-3-642-77011-1_10 1600751

[B6] Couedel-Courteille A. Butor C. Juillard V. Guillet J. G. Venet A. (1999). Dissemination of SIV after rectal infection preferentially involves paracolic germinal centers. Virology 260, 277–294 10.1006/viro.1999.9809 10417263

[B7] Couedel-Courteille A. Pretet J. L. Barget N. Jacques S. Petitprez K. Tulliez M. (2003). Delayed viral replication and CD4(+) T cell depletion in the rectosigmoid mucosa of macaques during primary rectal SIV infection. Virology 316, 290–301 10.1016/j.virol.2003.08.021 14644611

[B8] Delassus S. Cheynier R. Wain-Hobson S. (1992). Nonhomogeneous distribution of human immunodeficiency virus type 1 proviruses in the spleen. J. Virol. 66, 5642–5645 1501296 10.1128/jvi.66.9.5642-5645.1992 PMC289130

[B9] Desrosiers R. C. (1990). The simian immunodeficiency viruses. Annu. Rev. Immunol. 8, 557–578 10.1146/annurev.iy.08.040190.003013 2188674

[B10] Fujita Y. Otsuki H. Watanabe Y. Yasui M. Kobayashi T. Miura T. (2013). Generation of a replication-competent chimeric simian-human immunodeficiency virus carrying env from subtype C clinical isolate through intracellular homologous recombination. Virology 436, 100–111 10.1016/j.virol.2012.10.036 23219366

[B11] Fukazawa Y. Miyake A. Ibuki K. Inaba K. Saito N. Motohara M. (2008). Small intestine CD4+ T cells are profoundly depleted during acute simian-human immunodeficiency virus infection, regardless of viral pathogenicity. J. Virol. 82, 6039–6044 10.1128/JVI.02753-07 18400862 PMC2395167

[B12] Harouse J. M. Gettie A. Tan R. C. Blanchard J. Cheng-Mayer C. (1999). Distinct pathogenic sequela in rhesus macaques infected with CCR5 or CXCR4 utilizing SHIVs. Science 284, 816–819 10.1126/science.284.5415.816 10221916

[B13] Harouse J. M. Tan R. C. Gettie A. Dailey P. Marx P. A. Luciw P. A. (1998). Mucosal transmission of pathogenic CXCR4-utilizing SHIVSF33A variants in rhesus macaques. Virology 248, 95–107 10.1006/viro.1998.9236 9705259

[B14] Ho S. H. Shek L. Gettie A. Blanchard J. Cheng-Mayer C. (2005). V3 loop-determined coreceptor preference dictates the dynamics of CD4+-T-cell loss in simian-human immunodeficiency virus-infected macaques. J. Virol. 79, 12296–12303 10.1128/JVI.79.19.12296-12303.2005 16160156 PMC1211551

[B15] Horiike M. Iwami S. Kodama M. Sato A. Watanabe Y. Yasui M. (2012). Lymph nodes harbor viral reservoirs that cause rebound of plasma viremia in SIV-infected macaques upon cessation of combined antiretroviral therapy. Virology 423, 107–118 10.1016/j.virol.2011.11.024 22196013

[B16] Igarashi T. Brown C. R. Endo Y. Buckler-White A. Plishka R. Bischofberger N. (2001). Macrophage are the principal reservoir and sustain high virus loads in rhesus macaques after the depletion of CD4+ T cells by a highly pathogenic simian immunodeficiency virus/HIV type 1 chimera (SHIV): implications for HIV-1 infections of humans. Proc. Natl. Acad. Sci. U.S.A. 98, 658–663 10.1073/pnas.98.2.658 11136236 PMC14644

[B17] Igarashi T. Donau O. K. Imamichi H. Dumaurier M. J. Sadjadpour R. Plishka R. J. (2003). Macrophage-tropic simian/human immunodeficiency virus chimeras use CXCR4, not CCR5, for infections of rhesus macaque peripheral blood mononuclear cells and alveolar macrophages. J. Virol. 77, 13042–13052 10.1128/JVI.77.24.13042-13052.2003 14645561 PMC296065

[B18] Joag S. V. Adany I. Li Z. Foresman L. Pinson D. M. Wang C. (1997). Animal model of mucosally transmitted human immunodeficiency virus type 1 disease: intravaginal and oral deposition of simian/human immunodeficiency virus in macaques results in systemic infection, elimination of CD4+ T cells, and AIDS. J. Virol. 71, 4016–4023 9094679 10.1128/jvi.71.5.4016-4023.1997 PMC191554

[B19] Joag S. V. Li Z. Foresman L. Stephens E. B. Zhao L. J. Adany I. (1996). Chimeric simian/human immunodeficiency virus that causes progressive loss of CD4+ T cells and AIDS in pig-tailed macaques. J. Virol. 70, 3189–3197 8627799 10.1128/jvi.70.5.3189-3197.1996 PMC190182

[B20] Kodama T. Mori K. Kawahara T. Ringler D. J. Desrosiers R. C. (1993). Analysis of simian immunodeficiency virus sequence variation in tissues of rhesus macaques with simian AIDS. J. Virol. 67, 6522–6534 8411355 10.1128/jvi.67.11.6522-6534.1993 PMC238089

[B21] Kozyrev I. L. Ibuki K. Shimada T. Kuwata T. Takemura T. Hayami M. (2001). Characterization of less pathogenic infectious molecular clones derived from acute-pathogenic SHIV-89.6p stock virus. Virology 282, 6–13 10.1006/viro.2000.0839 11259185

[B22] Kuwata T. Byrum R. Whitted S. Goeken R. Buckler-White A. Plishka R. (2007). A rapid progressor-specific variant clone of simian immunodeficiency virus replicates efficiently *in vivo* only in the absence of immune responses. J. Virol. 81, 8891–8904 10.1128/JVI.00614-07 17596304 PMC1951398

[B23] Kuwata T. Igarashi T. Ido E. Jin M. Mizuno A. Chen J. (1995). Construction of human immunodeficiency virus 1/simian immunodeficiency virus strain mac chimeric viruses having vpr and/or nef of different parental origins and their *in vitro* and *in vivo* replication. J. Gen. Virol. 76, 2181–2191 10.1099/0022-1317-76-9-2181 7561755

[B24] Li Q. Duan L. Estes J. D. Ma Z. M. Rourke T. Wang Y. (2005). Peak SIV replication in resting memory CD4+ T cells depletes gut lamina propria CD4+ T cells. Nature 434, 1148–1152 15793562 10.1038/nature03513

[B25] Matsuda K. Inaba K. Fukazawa Y. Matsuyama M. Ibuki K. Horiike M. (2010). *In vivo* analysis of a new R5 tropic SHIV generated from the highly pathogenic SHIV-KS661, a derivative of SHIV-89.6. Virology 399, 134–143 10.1016/j.virol.2010.01.008 20102777

[B26] Mattapallil J. J. Douek D. C. Hill B. Nishimura Y. Martin M. Roederer M. (2005). Massive infection and loss of memory CD4+ T cells in multiple tissues during acute SIV infection. Nature 434, 1093–1097 10.1038/nature03501 15793563

[B27] Mehandru S. Poles M. A. Tenner-Racz K. Horowitz A. Hurley A. Hogan C. (2004). Primary HIV-1 infection is associated with preferential depletion of CD4+ T lymphocytes from effector sites in the gastrointestinal tract. J. Exp. Med. 200, 761–770 10.1084/jem.20041196 15365095 PMC2211967

[B28] Meng G. Sellers M. T. Mosteller-Barnum M. Rogers T. S. Shaw G. M. Smith P. D. (2000). Lamina propria lymphocytes, not macrophages, express CCR5 and CXCR4 and are the likely target cell for human immunodeficiency virus type 1 in the intestinal mucosa. J. Infect. Dis. 182, 785–791 10.1086/315790 10950772

[B29] Miyake A. Enose Y. Ohkura S. Suzuki H. Kuwata T. Shimada T. (2004). The quantity and diversity of infectious viruses in various tissues of SHIV-infected monkeys at the early and AIDS stages. Arch. Virol. 149, 943–955 10.1007/s00705-003-0252-0 15098109

[B30] Miyake A. Ibuki K. Enose Y. Suzuki H. Horiuchi R. Motohara M. (2006). Rapid dissemination of a pathogenic simian/human immunodeficiency virus to systemic organs and active replication in lymphoid tissues following intrarectal infection. J. Gen. Virol. 87, 1311–1320 10.1099/vir.0.81307-0 16603534

[B31] Motohara M. Ibuki K. Miyake A. Fukazawa Y. Inaba K. Suzuki H. (2006). Impaired T-cell differentiation in the thymus at the early stages of acute pathogenic chimeric simian-human immunodeficiency virus (SHIV) infection in contrast to less pathogenic SHIV infection. Microbes Infect. 8, 1539–1549 10.1016/j.micinf.2006.01.011 16702011

[B32] Mwimanzi P. Hasan Z. Hassan R. Suzu S. Takiguchi M. Ueno T. (2011). Effects of naturally-arising HIV Nef mutations on cytotoxic T lymphocyte recognition and Nef's functionality in primary macrophages. Retrovirology 8:50 10.1186/1742-4690-8-50 21696586 PMC3131245

[B33] Ndolo T. Syvanen M. Ellison T. Dandekar S. (2005). Evolution of nef variants in gut associated lymphoid tissue of rhesus macaques during primary simian immunodeficiency virus infection. Virology 343, 1–11 10.1016/j.virol.2005.08.013 16168456

[B34] Ono A. Freed E. O. (2004). Cell-type-dependent targeting of human immunodeficiency virus type 1 assembly to the plasma membrane and the multivesicular body. J. Virol. 78, 1552–1563 10.1128/JVI.78.3.1552-1563.2004 14722309 PMC321403

[B35] Oue M. Sakabe S. Horiike M. Yasui M. Miura T. Igarashi T. (2013). No viral evolution in the lymph nodes of simian immunodeficiency virus-infected rhesus macaques during combined antiretroviral therapy. J. Virol. 87, 4789–4793 10.1128/JVI.03367-12 23408611 PMC3624378

[B36] Overbaugh J. Rudensey L. M. Papenhausen M. D. Benveniste R. E. Morton W. R. (1991). Variation in simian immunodeficiency virus env is confined to V1 and V4 during progression to simian AIDS. J. Virol. 65, 7025–7031 1942255 10.1128/jvi.65.12.7025-7031.1991 PMC250821

[B37] Reimann K. A. Li J. T. Veazey R. Halloran M. Park I. W. Karlsson G. B. (1996). A chimeric simian/human immunodeficiency virus expressing a primary patient human immunodeficiency virus type 1 isolate env causes an AIDS-like disease after *in vivo* passage in rhesus monkeys. J. Virol. 70, 6922–6928 8794335 10.1128/jvi.70.10.6922-6928.1996 PMC190741

[B38] Reimann K. A. Watson A. Dailey P. J. Lin W. Lord C. I. Steenbeke T. D. (1999). Viral burden and disease progression in rhesus monkeys infected with chimeric simian-human immunodeficiency viruses. Virology 256, 15–21 10.1006/viro.1999.9632 10087222

[B39] Ritola K. Pilcher C. D. Fiscus S. A. Hoffman N. G. Nelson J. A. Kitrinos K. M. (2004). Multiple V1/V2 env variants are frequently present during primary infection with human immunodeficiency virus type 1. J. Virol. 78, 11208–11218 10.1128/JVI.78.20.11208-11218.2004 15452240 PMC521858

[B40] Scharko A. M. Perlman S. B. Hinds P. W. 2nd. Hanson J. M. Uno H. Pauza C. D. (1996). Whole body positron emission tomography imaging of simian immunodeficiency virus-infected rhesus macaques. Proc. Natl. Acad. Sci. U.S.A. 93, 6425–6430 10.1073/pnas.93.13.6425 8692831 PMC39039

[B41] Scharko A. M. Perlman S. B. Pyzalski R. W. Graziano F. M. Sosman J. Pauza C. D. (2003). Whole-body positron emission tomography in patients with HIV-1 infection. Lancet 362, 959–961 10.1016/S0140-6736(03)14366-8 14511930

[B42] Sharova N. Swingler C. Sharkey M. Stevenson M. (2005). Macrophages archive HIV-1 virions for dissemination in trans. EMBO J. 24, 2481–2489 10.1038/sj.emboj.7600707 15920469 PMC1173148

[B43] Shinohara K. Sakai K. Ando S. Ami Y. Yoshino N. Takahashi E. (1999). A highly pathogenic simian/human immunodeficiency virus with genetic changes in cynomolgus monkey. J. Gen. Virol. 80, 1231–1240 10355770 10.1099/0022-1317-80-5-1231

[B44] Suryanarayana K. Wiltrout T. A. Vasquez G. M. Hirsch V. M. Lifson J. D. (1998). Plasma SIV RNA viral load determination by real-time quantification of product generation in reverse transcriptase-polymerase chain reaction. AIDS Res. Hum. Retroviruses 14, 183–189 10.1089/aid.1998.14.183 9462929

[B45] Thompson J. D. Gibson T. J. Plewniak F. Jeanmougin F. Higgins D. G. (1997). The CLUSTAL_X windows interface: flexible strategies for multiple sequence alignment aided by quality analysis tools. Nucleic Acids Res. 25, 4876–4882 10.1093/nar/25.24.4876 9396791 PMC147148

[B46] Veazey R. S. DeMaria M. Chalifoux L. V. Shvetz D. E. Pauley D. R. Knight H. L. (1998). Gastrointestinal tract as a major site of CD4+ T cell depletion and viral replication in SIV infection. Science 280, 427–431 10.1126/science.280.5362.427 9545219

[B47] Veazey R. S. Mansfield K. G. Tham I. C. Carville A. C. Shvetz D. E. Forand A. E. (2000). Dynamics of CCR5 expression by CD4(+) T cells in lymphoid tissues during simian immunodeficiency virus infection. J. Virol. 74, 11001–11007 10.1128/JVI.74.23.11001-11007.2000 11069995 PMC113180

[B48] Wong J. K. Ignacio C. C. Torriani F. Havlir D. Fitch N. J. Richman D. D. (1997). *In vivo* compartmentalization of human immunodeficiency virus: evidence from the examination of pol sequences from autopsy tissues. J. Virol. 71, 2059–2071 9032338 10.1128/jvi.71.3.2059-2071.1997 PMC191294

